# Open questions in chemistry

**DOI:** 10.1038/s42004-020-00351-8

**Published:** 2020-08-07

## Abstract

Today, *Communications Chemistry* launches a series of Comment articles discussing key open questions in specific fields of fundamental chemical research. Here we outline the aims of this series and highlight each contribution within.

In spite of decades of research and the enormous progress made, chemists continue to grapple with poorly understood aspects of the world around us. Indeed, many fundamental questions remain—will we ever get to the bottom of the different structures of ice, or pinpoint the origins of life on Earth, or obtain a full picture of the complexity of carbon-based molecules in space?

This series aims to uncover open questions across the breadth of chemistry. Each Comment provides an overview of a focused field of research, identifies key open questions, and gives expert opinion on how challenges in answering these questions might be overcome.

Our hope is that this series will be of interest to the chemistry community as a whole, and that it will stimulate those working in or adjacent to the research fields that are covered to think about some of the open questions that invite further study. From chemical biology to physical chemistry, the following contributions mark the launch of this series.

Ben Schummann and Mia Zol-Hanlon (The Francis Crick Institute, UK) describe the challenges faced by chemical glycobiologists (10.1038/s42004-020-00337-6). Although all cells and most proteins are decorated with carbohydrates called glycans, our ability to characterise the structure and function of these intriguing molecules is hindered by biology and technology alike. Schumann and Zol-Hanlon outline key challenges to our understanding of the chemical biology of glycans, and discuss the opportunities offered by chemical methodologies to deepen our insight into this exciting frontier.

Debbie Crans and Kateryna Kostenkova (Colorado State University, USA) outline open questions on the biological roles of the first-row transition metals (10.1038/s42004-020-00341-w). These elements present sensitive biochemistry, and while most play important roles in biological processes and in medicine, they can become toxic at high concentrations. Crans and Kostenkova highlight the importance of furthering our understanding of their speciation chemistry, coordination chemistry, metal transfer reactions, and bioprocessing and metabolism.

Rebecca Abergel and colleagues (University of California, Berkeley, USA) explain that, owing to their scarcity and inherent radioactivity, our understanding of the structure, bonding and reactivity of the transplutionium elements is relatively poorly developed (10.1038/s42004-020-00338-5). They outline how recent advances in spectroscopic and computational tools have nevertheless facilitated the study of transplutonium coordination chemistry, and discuss whether furthering our knowledge of covalency and heterogeneity changes in 5*f* orbital bonding could be harnessed in environmentally and industrially relevant systems.

Taina Yli-Juuti (University of Eastern Finland, Finland) and colleagues from Stockholm University, Sweden comment on the condensational growth of nucleated atmospheric particles to sizes where they influence cloud formation (10.1038/s42004-020-00339-4). They highlight that our knowledge of the properties of the vapours that grow atmospheric particles is still incomplete, and that global and regional scale models to describe particle growth are needed to improve our understanding of aerosol–cloud interactions, which are one of the most uncertain aspects of anthropogenic climate forcing.

Barbara Finlayson-Pitts and team (University of California, Irvine, USA) give an overview of the chemical composition of aerosols, noting how particle composition, size, and phase are intricately linked (10.1038/s42004-020-00347-4). They describe how far analytical techniques have come in unveiling the chemical composition of airborne particles, and highlight the challenges that researchers face in reliably mapping atmospheric particle composition. The team remark that methods that do not alter sample composition during analysis will be key.

Bryan Bzdek, Jonathan Reid and Michael Cotterell (University of Bristol, UK) elaborate on another aspect of aerosol science that poses a number of open questions: their physical properties (10.1038/s42004-020-00342-9). Aerosols are highly dynamic, non-equilibrium systems with very different microphysical properties than bulk systems. The team comment on aerosol optical properties, and on how their physical properties affect (bio)chemical transformations, reaction rates, and even microorganism survival.

Thomas Lörting and colleagues (University of Innsbruck, Austria) guide the reader through a number of known and predicted crystalline water ice structures (10.1038/s42004-020-00349-2). From negative to ultra-high pressure realms, they comment on hydrogen bonding as well as geometry-frustrated ices. The group points to open questions on the structures of water ices, including even at ambient pressure conditions, and speculate on possible future discoveries.

Massimiliano Esposito (University of Luxembourg, Luxembourg) surveys the advances made in extending theories of non-equilibrium thermodynamics to the analysis of chemical reaction networks (10.1038/s42004-020-00344-7). Non-equilibrium systems are of increasing interest among experimental chemists, in part because they may offer minimal models of biological machines and networks. Esposito outlines the substantial theoretical progress made in recent decades and the key challenges that currently limit our ability to characterise the thermodynamics of complex chemical reaction networks such as metabolic pathways.

While the above Comments delve into a number of important and exciting topics, when one considers the breadth of ongoing research in fundamental areas of chemistry, we have only just begun to scratch the surface. We look forward to publishing more in this series in the months and years to come, and invite experts to contact us if they are interested in contributing. We would be particularly pleased to hear from those from underrepresented minority groups, and will strive to ensure that the series is representative of all voices of the chemistry community as it continues to develop.

